# Streptococcus pneumoniae Binds to Host Lactate Dehydrogenase via PspA and PspC To Enhance Virulence

**DOI:** 10.1128/mBio.00673-21

**Published:** 2021-05-04

**Authors:** Sang-Sang Park, Norberto Gonzalez-Juarbe, Eriel Martínez, Joanetha Yvette Hale, Yi-Han Lin, Joshua T. Huffines, Katherine L. Kruckow, David E. Briles, Carlos J. Orihuela

**Affiliations:** a Department of Microbiology, The University of Alabama at Birmingham, Birmingham, Alabama, USA; b Infectious Diseases and Genomic Medicine Group, J Craig Venter Institute, Rockville, Maryland, USA; Carnegie Mellon University

**Keywords:** pneumococcal surface protein A, pneumococcal surface protein C, *Streptococcus pneumoniae*, choline binding proteins, pathogenesis, pneumonia, virulence

## Abstract

Pneumococcal surface protein A (PspA) and pneumococcal surface protein C (PspC, also called CbpA) are major virulence factors of Streptococcus pneumoniae (*Spn*). These surface-exposed choline-binding proteins (CBPs) function independently to inhibit opsonization, neutralize antimicrobial factors, or serve as adhesins. PspA and PspC both carry a proline-rich domain (PRD) whose role, other than serving as a flexible connector between the N-terminal and C-terminal domains, was up to this point unknown. Herein, we demonstrate that PspA binds to lactate dehydrogenase (LDH) released from dying host cells during infection. Using recombinant versions of PspA and isogenic mutants lacking PspA or specific domains of PspA, this property was mapped to a conserved 22-amino-acid nonproline block (NPB) found within the PRD of most PspAs and PspCs. The NPB of PspA had specific affinity for LDH-A, which converts pyruvate to lactate. In a mouse model of pneumonia, preincubation of *Spn* carrying NPB-bearing PspA with LDH-A resulted in increased bacterial titers in the lungs. In contrast, incubation of *Spn* carrying a version of PspA lacking the NPB with LDH-A or incubation of wild-type *Spn* with enzymatically inactive LDH-A did not enhance virulence. Preincubation of NPB-bearing *Spn* with lactate alone enhanced virulence in a pneumonia model, indicating exogenous lactate production by *Spn*-bound LDH-A had an important role in pneumococcal pathogenesis. Our observations show that lung LDH, released during the infection, is an important binding target for *Spn* via PspA/PspC and that pneumococci utilize LDH-A derived lactate for their benefit *in vivo*.

## INTRODUCTION

Streptococcus pneumoniae (*Spn*) is an opportunistic human pathogen capable of causing a wide spectrum of serious infectious diseases, including pneumonia, bacteremia, sepsis, and meningitis (1). The pneumococcus is the leading cause of community-acquired pneumonia and invasive disease worldwide (2). *Spn* is a Gram-positive bacterium with teichoic acid and lipoteichoic acid being major constituents of its cell wall and cell membrane, respectively (3). In *Spn*, and some but not all oral streptococci, these glycopolymers contain phosphorylcholine residues which serve as anchors for a family of surface proteins known as choline-binding proteins (CBPs). CBPs bind noncovalently to the phosphorylcholine moieties of teichoic and lipoteichoic acids via a choline-binding domain (CBD) composed of repeating, conserved choline-binding motifs at either their N or C terminus. CBPs play diverse and vital roles in the biology of the pneumococcus and are involved in cell wall remodeling, autolysis, biofilm formation, host cell adhesion and invasion, and resistance to antimicrobial factors such as complement and cationic antimicrobial peptides (4).

**FIG 1:**
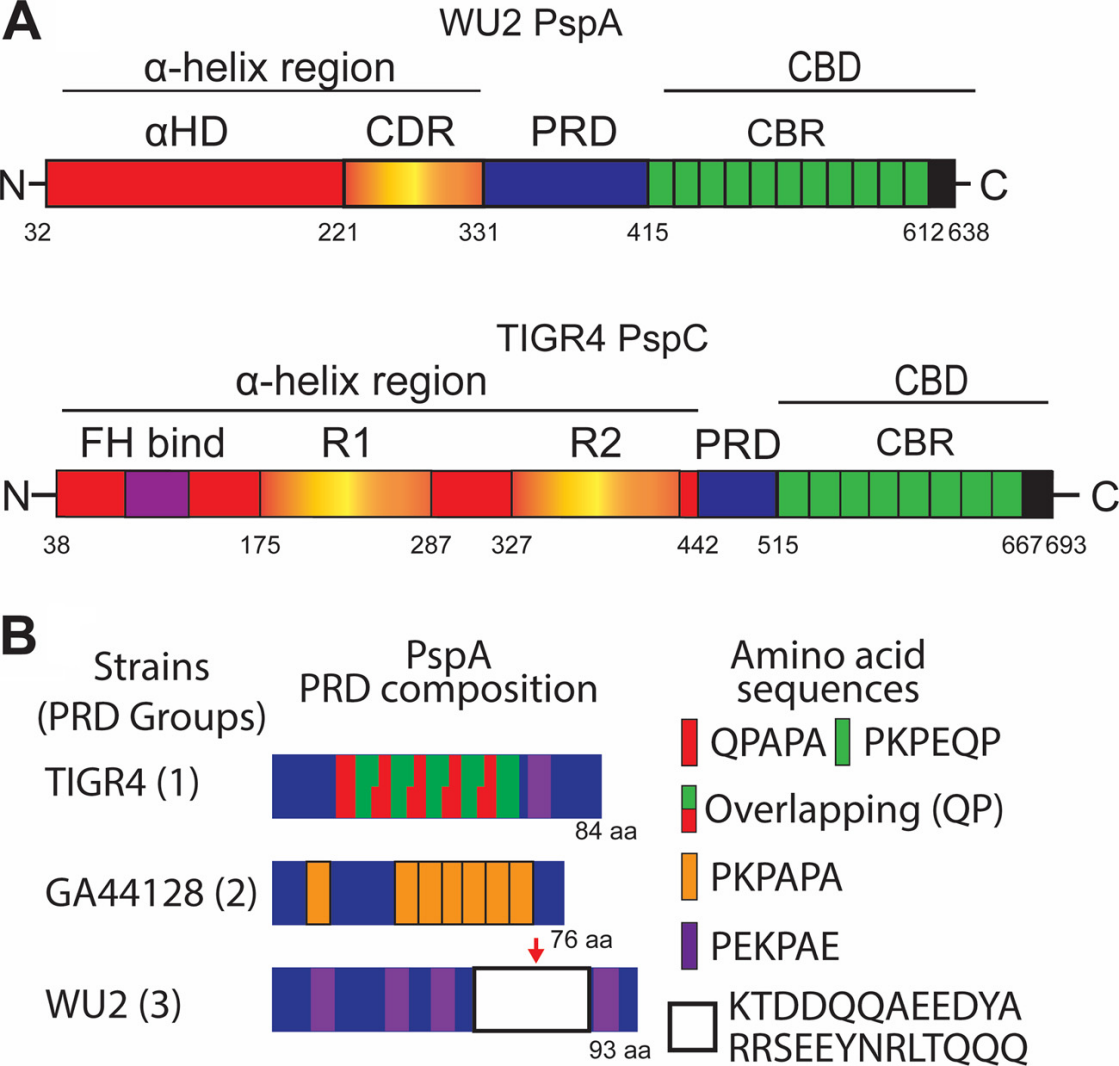
Schematic diagrams of PspA, PspC, and representative proline-rich domains. (A) Schematic diagram of serotype 3 strain WU2 PspA and serotype 4 strain TIGR4 PspC. Shown for PspA is the N-terminal α-helix region, composed of the α-helix domain (αHD) and clade defining region (CDR), the proline-rich domain (PRD), and C-terminal choline-binding domain (CBD) composed of multiple choline-binding repeats (CBR). The α-helix region of PspC is composed of the factor H binding region (FH bind), two R domains (R1 and R2), and the PRD. This is followed by the CBD. (B) Schematic diagrams of PRDs from group 1, 2, and 3. Groups 1 and 2 are composed of different proline-repeating sequences. Group 3 shares aspects of both group 1 and group 2 but is characterized by the presence of the nonproline block (NPB). The red arrow indicates the NPB.

Pneumococcal surface protein A (PspA) and pneumococcal surface protein C (PspC; also known as CbpA) are among the most investigated CBPs (5, 6). PspA and PspC have loosely similar structural organizations with their N termini composed of a series of α-helical domains (Fig. 1A). PspA ranges from 65 to 95 kDa in size with an N-terminal signal peptide, followed by an α-helix domain (αHD) which contains what is known as the clade defining region (CDR). Next is the proline-rich domain (PRD), which is often interrupted by a highly conserved 22-amino-acid sequence called the nonproline block (NPB). At the C terminus is the CBD (6). PspC ranges from 59 to 105 kDa in size and also has an N-terminal signal peptide followed by a long multifunctional module that contains a factor H binding motif and two repeats of an R domain (R1 and R2), each of which is a three-α-helical raft-like structure (7). These are followed by a PRD and then the CBD (5, 6).

PspA has been demonstrated to bind to and prevent killing by the antimicrobial peptide lactoferricin. It has also been shown to block complement-activating host C-reactive protein from binding to exposed, i.e., unoccupied, phosphorylcholine residues on the bacterial cell wall (8, 9). PspC binds to polymeric immunoglobulin receptors and laminin receptors on host cells. These interactions have been shown to be responsible for *Spn* translocation across mucosal epithelial and vascular endothelial cell barriers, respectively (10, 11). Some, but not all, PspCs also bind serum factor H. The binding of factor H helps protect pneumococci against complement deposition on the bacterial surface (12, 13). Pertinent to this study, the role of PRD on either PspA or PspC was unknown, although the diversity of this domain had been explored for PspA since it has been shown to elicit protective antibody (14, 15). In 2018, we showed that PRD in PspA of different *Spn* isolates can be divided into 3 groups based on its amino acid sequence (14). Group 1 and 2 are divided based upon the arrangement of highly conserved proline-rich amino acid sequences. Group 3 shares aspects of both group 1 and group 2, but group 3 proline-rich sequences are interrupted by a highly conserved 22-amino-acid sequence called the highly conserved nonproline block (NPB) (sequence provided in Fig. 1B). Notably, ∼90% of *Spn* isolates have a version of PspA (48% to 56% of 123 strains) or PspC (77% of 43 strains) that is interrupted by the NPB (5, 6, 14–16), but a role for this sequence motif remained undiscovered.

Lactate dehydrogenase (LDH) is a key enzyme involved in cellular metabolism and is one of the most highly expressed proteins in mammalian cells. Mammalian LDH is a tetramer composed of LDH-A and/or LDH-B. The specific ratio of LDH-A to LDH-B is dependent on the cell type; in lung epithelial cells, they are in equal amounts (17–19). LDH-A converts pyruvate to lactate and generates NAD^+^ from NADH, whereas LDH-B converts lactate to pyruvate and reduces NAD^+^ to create NADH (20). *Spn* produces a variety of molecules that have been demonstrated to be cytotoxic to host cells. Primary among these are pneumolysin (Ply), a pore-forming toxin, and H_2_O_2_, which is produced by the enzyme streptococcal pyruvate oxidase (SpxB) as part of the bacterium’s own metabolism (21–23). During pneumococcal pneumonia, cytoplasmic LDH is released from dying host cells as result of exposure to Ply, SpxB-derived H_2_O_2_, and other cytotoxic molecules produced by the bacteria. Indeed, the amount of LDH present in tissue culture supernatants is frequently used as a measure of host cell death (22).

In this paper, we show that the NPB of both of PspA and PspC binds to mammalian LDH-A. Moreover, the generation of lactate by host LDH-A directly enhances the virulence of *Spn* as result of co-opted metabolic activity. Our results add to the increasing body of work that shows *Spn* appropriates host factors released by dying cells to exacerbate its virulence.

## RESULTS

### *Spn* binds to LDH released from dying cells.

**FIG 2:**
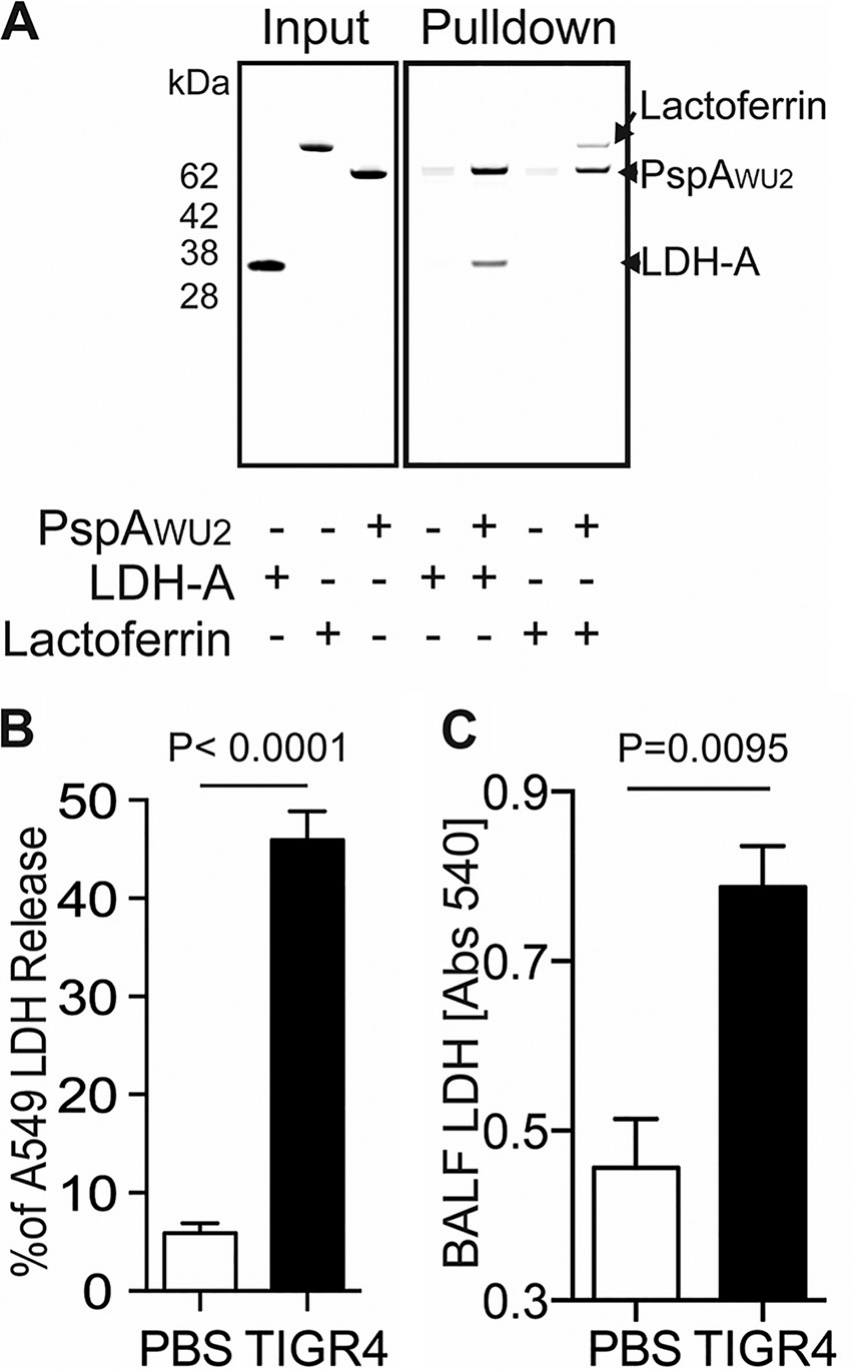
PspA binds to LDH from dying host cell via *Spn* infection. (A) Recombinant His-tagged PspA_WU2_ protein was pulled down with purified host LDH-A and human lactoferrin. The bound proteins were separated by SDS-PAGE and visualized by Coomassie blue dye. This experiment was performed two times. (B) Human A549 type II pulmonary epithelial cells were infected with *Spn* strain TIGR4 for 4 h at an MOI of 100. Cultured medium was collected to measure percent LDH release when normalized to that from uninfected cells and total lysis using lysis buffer for 30 min. (C) Six- to eight-week-old C57BL/6 mice were challenged intratracheally with *Spn* TIGR4 (5 × 10^5^ CFU) or mock infected with PBS. One day after pneumococcal challenge, relative levels of LDH in bronchoalveolar lavage fluid (BALF) samples were determined by ELISA. Means and standard errors are shown. Statistical analyses were performed using a Mann-Whitney U test.

Given that PspA is a highly abundant surface protein, we explored the possibility that it modulated *Spn* virulence in a yet-unknown fashion. To identify previously unknown host proteins that PspA interacted with, we performed pulldown experiments using His-tagged recombinant PspA from strain WU2 (PspA_WU2_), composed of poly-His-tagged PspA αHD and PRD, as bait for proteins in THP-1 human monocyte cell lysates. Among the top five host proteins identified following separation of eluted proteins by SDS-PAGE, band excision, and identification by mass spectroscopy was lactate dehydrogenase A (LDH-A) (see Fig. S1 and Table S1 in the supplemental material). LDH-A was particularly intriguing given that PspA also bound to LDH when tested by enzyme-linked immunosorbent assay (ELISA) using cell lysates from A549 human type II pneumocytes (see Fig. S2A) and because LDH is known to be released by dying host cells as result of *Spn* infection (22). Notably, and in comparison, PspA bound poorly to pneumococcal LDH as determined by ELISA (Fig. S2B). To further validate the interaction of PspA with host LDH, we repeated pulldown experiments using purified rabbit muscle LDH, consisting primarily of LDH-A, and purified lactoferrin, since it is known to bind PspA (9). PspA_WU2_ bound to a nickel column pulled down both these proteins, whereas they were not pulled down when PspA_WU2_ was absent (Fig. 2A). We also reaffirmed that host LDH was released from human lung cells *in vitro* and *in vivo* into the bronchi of mice following challenge or the induction of *Spn* pneumonia, respectively (Fig. 2B and C). Thus, PspA binds the bacterium to host LDH, which becomes accessible during the course of infection.

10.1128/mBio.00673-21.1 TABLE S1 List of PspA interacted THP-1 proteins. Download Table S1, PDF file, 0.02 MB. Copyright © 2021 Park et al. 2021 Park et al. https://creativecommons.org/licenses/by/4.0/ This content is distributed under the terms of the Creative Commons Attribution 4.0 International license.

10.1128/mBio.00673-21.3 FIG S1 Purified PspA binds to host LDH and glyceraldehyde-3-phosphate dehydrogenase (GAPDH). Recombinant His-tagged pneumolysin (Ply, green arrowhead) or PspA_WU2_ (yellow arrowhead) were pulled down with human macrophage THP-1 cell lysate using Ni-NTA resin. The input and bound proteins were separated by SDS-PAGE and visualized by Coomassie blue dye. PspA bound host proteins (red arrowheads) are listed on Table S1. Download FIG S1, PDF file, 1.0 MB. Copyright © 2021 Park et al. 2021 Park et al. https://creativecommons.org/licenses/by/4.0/ This content is distributed under the terms of the Creative Commons Attribution 4.0 International license.

10.1128/mBio.00673-21.4 FIG S2 PspA interaction with host and Spn LDH. (A) PspA binds to A549 type II pneumocyte cell line LDH. Interaction of PspA with A549 LDH was analyzed by ELISA using recombinant PspA_WU2_. (B) PspA has low affinity for *Spn* LDH compared to host LDH. Means and standard errors shown. Statistical analyses were performed using a *t* test. Download FIG S2, PDF file, 0.06 MB. Copyright © 2021 Park et al. 2021 Park et al. https://creativecommons.org/licenses/by/4.0/ This content is distributed under the terms of the Creative Commons Attribution 4.0 International license.

### PspA binds to LDH-A via the NPB.

**FIG 3:**
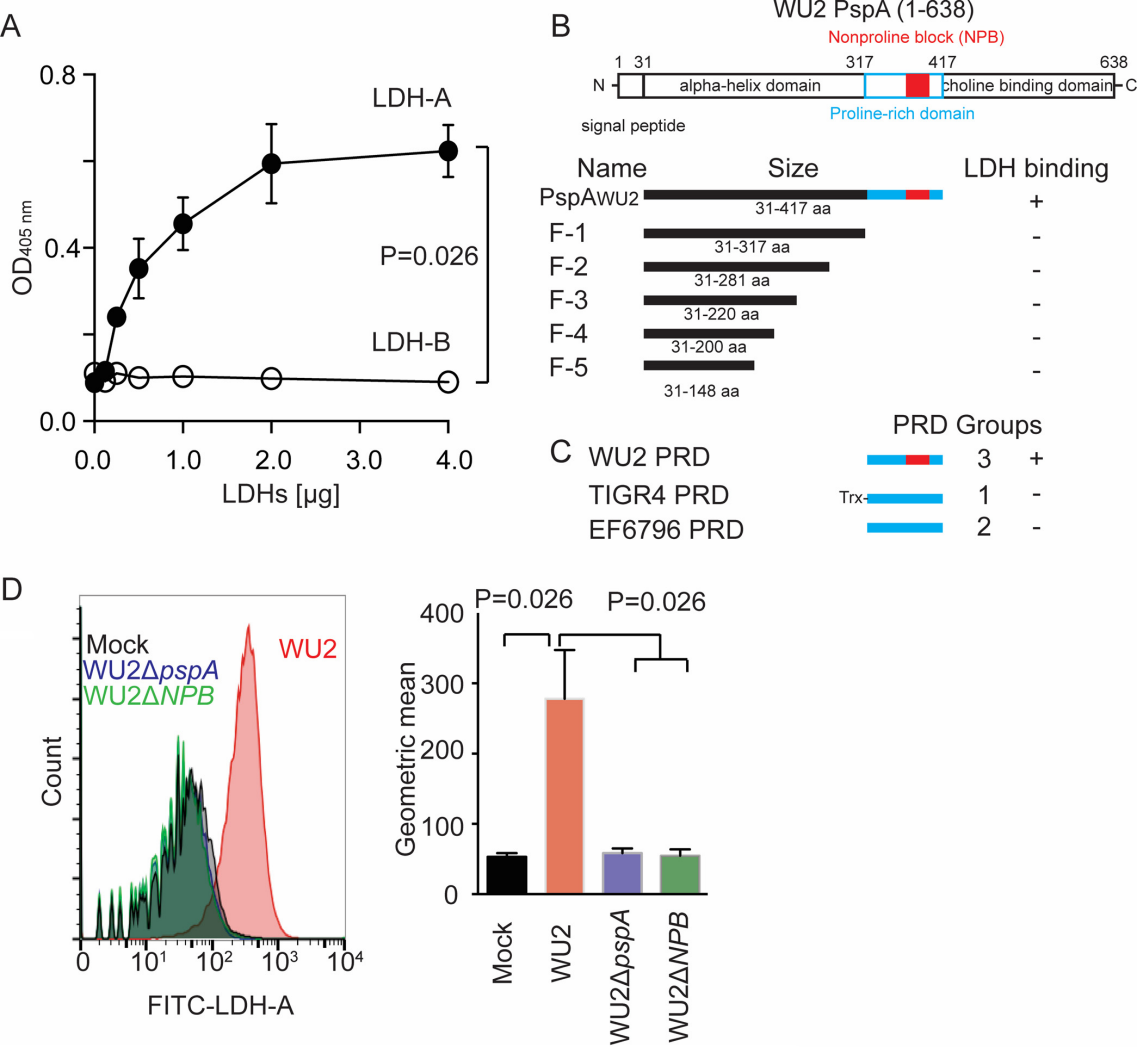
PspA binds to LDH-A via NPB. (A) Interaction of PspA with LDH-A or LDH-B was analyzed by ELISA under different PspA_WU2_ concentrations. (B) Diagram of PspA_WU2_ fragments (F-1 to F-5) used in the pulldown experiments, the corresponding truncated mutants created for testing of PspA binding to LDH-A and PRDs (see Fig. S3A in the supplemental material). (C) Different versions of PRD from designated *Spn* strains were tested for their ability to bind LDH-A in pulldown experiments (Fig. S3B). (D) *Spn* WU2, its isogenic *pspA* deletion mutant (WU2ΔpspA), and a isogenic NPB deletion mutant of PspA (WU2ΔNPB) were incubated with FITC-conjugated rabbit LDH-A. The LDH-A binding to pneumococci was measured by flow cytometry. Data are geometric means (means and standard errors shown) of the log fluorescence intensity measured on a flow cytometer (*n* = 3).

LDH tetramers within lung epithelial cells are composed equally of LDH-A and LDH-B (24). To determine if PspA had affinity for LDH-A, LDH-B, or both, we performed ELISAs with recombinant purified human LDH-A and LDH-B. Strong dose-response binding of LDH-A and PspA_WU2_ was observed, whereas LDH-B showed no binding (Fig. 3A). To identify the LDH-binding domain of PspA, a second series of pulldown assays was performed with increasingly smaller fragments (F) of PspA_WU2_: F-1, F-2, F-3, F-4, and F-5. Only full-length PspA_WU2_ (composed of αHD and PRD) pulled down LDH-A, suggesting that PRD was the responsible domain (Fig. 3B; see also Fig. S3A). These experiments were next repeated with recombinant PspA PRDs from strain TIGR4 (PRD group 1), strain EF6796 (PRD group 2), and WU2 (PRD group 3). Only WU2 PRD, which contains the NPB, captured LDH-A (Fig. 3C; Fig. S3B). Critically, we observed that wild-type WU2, but not an isogenic mutant deficient in PspA (WU2 Δ*pspA*) or an isogenic WU2 mutant carrying PspA with the NPB genetically deleted from the PRD (WU2 Δ*pspA-NPB*), bound to LDH-A in flow cytometry experiments (Fig. 3D). Thus, our collective experimental results indicate that the NPB within the PRD of PspA was responsible for specific binding to LDH-A.

10.1128/mBio.00673-21.5 FIG S3 LDH binds to group 3 PRD motif. PspA_WU2_, fragments (F-1, F-2, F-3, F-4, and F-5) (A) and PRDs (WU2 PRD, EF6978 PRD and TIGR4 PRD) as bait (B) were pulled down with LDH and as visualized by Coomassie blue stains. Bound LDH were detected by immunoblotting using monoclonal anti-LDH antibody. The red arrows indicate LDH. Download FIG S3, PDF file, 0.6 MB. Copyright © 2021 Park et al. 2021 Park et al. https://creativecommons.org/licenses/by/4.0/ This content is distributed under the terms of the Creative Commons Attribution 4.0 International license.

### NPB on PspC also binds to LDH-A.

**FIG 4:**
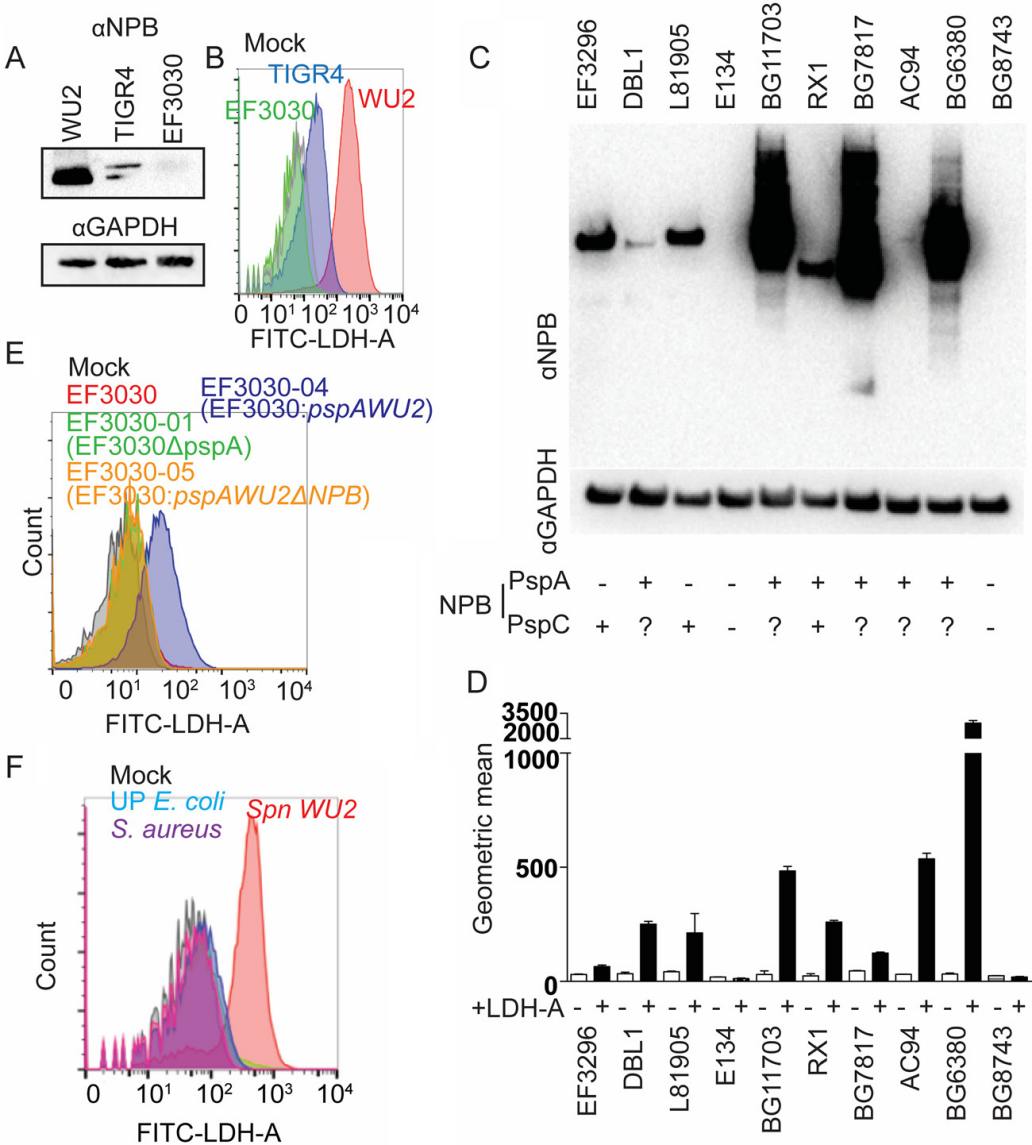
NPB on PspA and PspC binds to LDH. (A) Immunoblots of total bacterial cell lysates using anti-NPB monoclonal antibody PR-1A4.7 with total bacterial cell lysate was used to detect the NPB domain in WU2, TIGR4, and EF3030. (B) *Spn* WU2, TIGR4, or EF3030 was incubated with FITC-conjugated LDH. LDH binding to pneumococci was measured using flow cytometry. This experiment was performed two times with identical results. (C) Immunoblots using NPB monoclonal antibody of total *Spn* bacterial cell lysates from designated clinical isolates. The presence of PspA or PspC containing the NPB as inferred from publicly available genomic sequence data is indicated on the bottom. (D) The ability of these isolates to bind rabbit LDH-A was also measured using flow cytometry (*n* = 3). (E) EF3030, EF3030 isogenic *pspA* mutant (EF3030-01), EF3030 expressing PspA_WU2_ (EF3030-04), and EF3030 expressing PspA_WU2_ without NPB (EF3030-05) were incubated with FITC-conjugated rabbit LDH-A. The LDH-A binding to pneumococci was measured by flow cytometry. (F) *Spn* WU2, S. aureus, and urinary tract-pathogenic E. coli (UP E. coli) were incubated with FITC-conjugated rabbit LDH-A. The LDH binding to pneumococci was measured by flow cytometry. This experiment was performed two times with similar results.

As indicated, recombinant PRD from TIGR4 PspA did not interact with LDH-A (Fig. 3C). Yet, the annotated genome of TIGR4 indicates this strain carries a version of PspC with a group 3 PRD containing the NPB, suggesting TIGR4 should have the capacity to bind LDH-A. As expected, the NPB motif was detected in TIGR4 cell lysates by immunoblot using monoclonal antibody reactive with NPB (Fig. 4A). Furthermore, live TIGR4 was observed to bind LDH-A by flow cytometry (Fig. 4B). Notably, this was also not the case for EF3030, a serotype 19F strain whose versions of PspA and PspC do not have the NPB domain (Fig. 4A and B). Subsequent examination of a panel of 10 *Spn* clinical isolates showed that our detection of the NPB in bacterial lysates using monoclonal antibody via immunoblot was consistently positively correlated with LDH-A binding capacity of live pneumococci (Fig. 4C and D). Of note, and in contrast to other *Spn* strains, WU2 is unusual and does not carry PspC (5). Thus, our prior results with WU2 were fortuitously not confounded by the presence of a second PRD. To reaffirm that it was the NPB within the PRD that was responsible, EF3030 was mutated so as to carry distinct versions of PspA. Only the version of EF3030 expressing PspA carrying NPB within the PRD showed LDH binding by flow cytometry (Fig. 4E; see also Fig. S4). Finally, we incubated fluorescein isothiocyanate (FITC)-conjugated LDH-A with WU2 and clinical isolates of Staphylococcus aureus and uropathogenic Escherichia coli and then analyzed LDH-A binding ability by flow cytometry. Only WU2 showed affinity for LDH-A (Fig. 4F). Thus, LDH-A binding is not shared by other major pathogens.

10.1128/mBio.00673-21.6 FIG S4 PspA patterns of WU2 and EF3030 wild type or different isogenic mutants. Total cell lysates of *Spn* WU2, EF3030, isogenic *pspA* mutants (WU2Δ*pspA* and EF3030-01), isogenic pspA mutants (WU2Δ*NPB*), EF3030 expressing PspA_WU2_ (EF3030-04), and EF3030 expressing PspA_WU2_ without NPB (EF3030-05) were electrophorized on SDS-PAGE gels. Subsequently, expressed PspA were detected by using monoclonal anti-PspA antibody. Download FIG S4, PDF file, 0.3 MB. Copyright © 2021 Park et al. 2021 Park et al. https://creativecommons.org/licenses/by/4.0/ This content is distributed under the terms of the Creative Commons Attribution 4.0 International license.

### LDH binding to pneumococci enhances *Spn* virulence *in vitro*.

**FIG 5:**
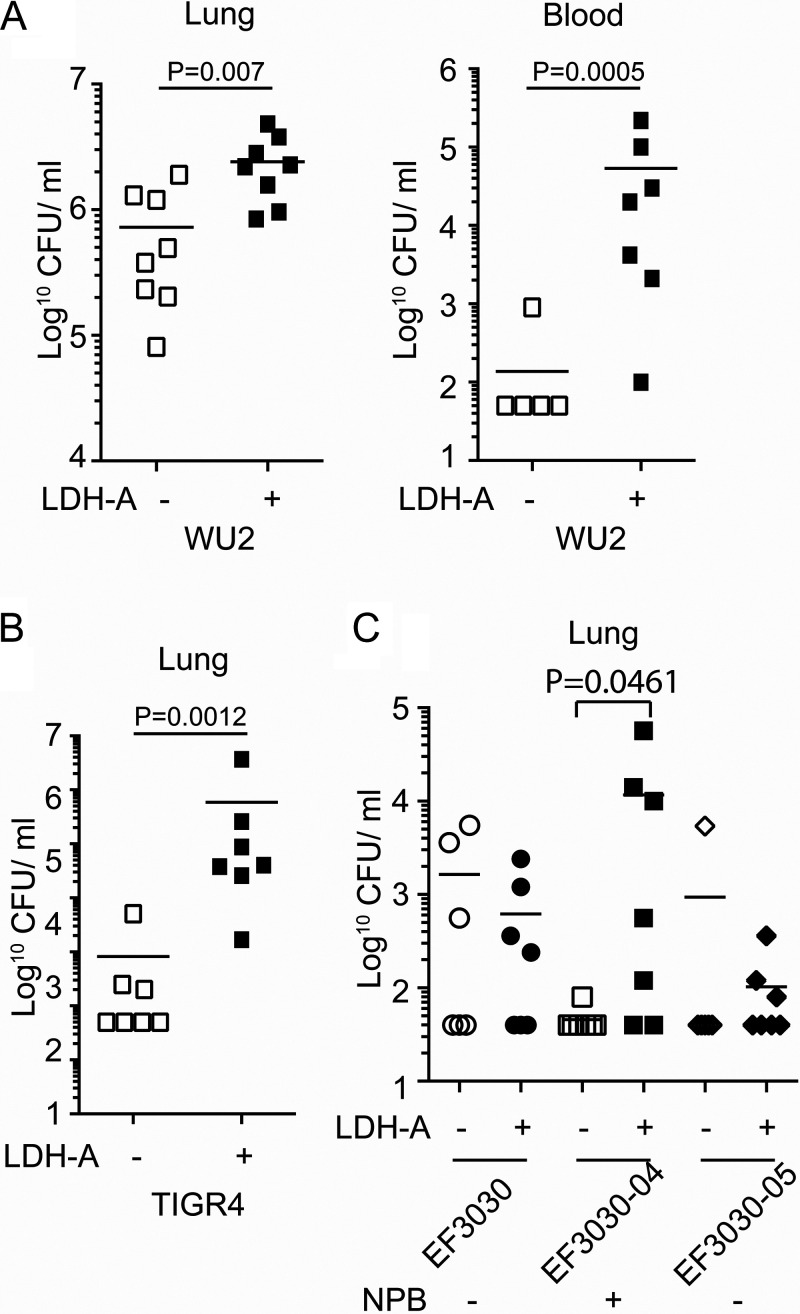
LDH-A enhanced *Spn* pathogenesis. Mice were infected (A) intratracheally with 5.0 × 10^6^ CFU of WU2 or (B) 1.0 × 10^5^ CFU of TIGR4 following coincubation and alongside rabbit LDH-A. (C) Mice were infected intratracheally with *Spn* preincubated with rabbit LDH-A. Mice received 1.0 × 10^6^ CFU of EF3030, EF3030-04, or EF3030-05 intratracheally. Shown are bacterial titers in the lungs and blood 24 h after challenge. Shapes in panels A, B, and C represent bacterial burden for each mouse. Statistical analyses were performed using a Mann-Whitney U test or Kruskal-Wallis test with two-way ANOVA.

The prevalence of the PRD containing NPB in both PspA and PspC, both of which are vital virulence determinants, suggests the NPB of PspC also contributes to *Spn* virulence. To test this, we challenged mice with WU2 or TIGR4. WU2 has NPB in PspA but does not have PspC. TIGR4 has an NPB in its PspC but not in its PspA. In each case, the inoculum was given in phosphate-buffered saline (PBS) with or without rabbit LDH-A. Strikingly, mixture of these bacteria with LDH-A prior to challenge resulted in >10-fold more pneumococci in the lungs of mice within a 24-h period and a greater likelihood of developing bacteremia (Fig. 5A and B). We also examined the virulence of EF3030 carrying versions of PspA with and without the NPB. EF3030 carrying PspA with the NPB was more virulent following bacterial mixture with LDH-A, whereas no LDH-A enhancement of virulence was observed for EF3030 with PspA lacking NPB (Fig. 5C). We conclude host LDH-A binding to PspA or PspC, via the NPB, enhanced the virulence of *Spn*.

### LDH-A enzymatic activity is required for *Spn* virulence enhancement.

**FIG 6:**
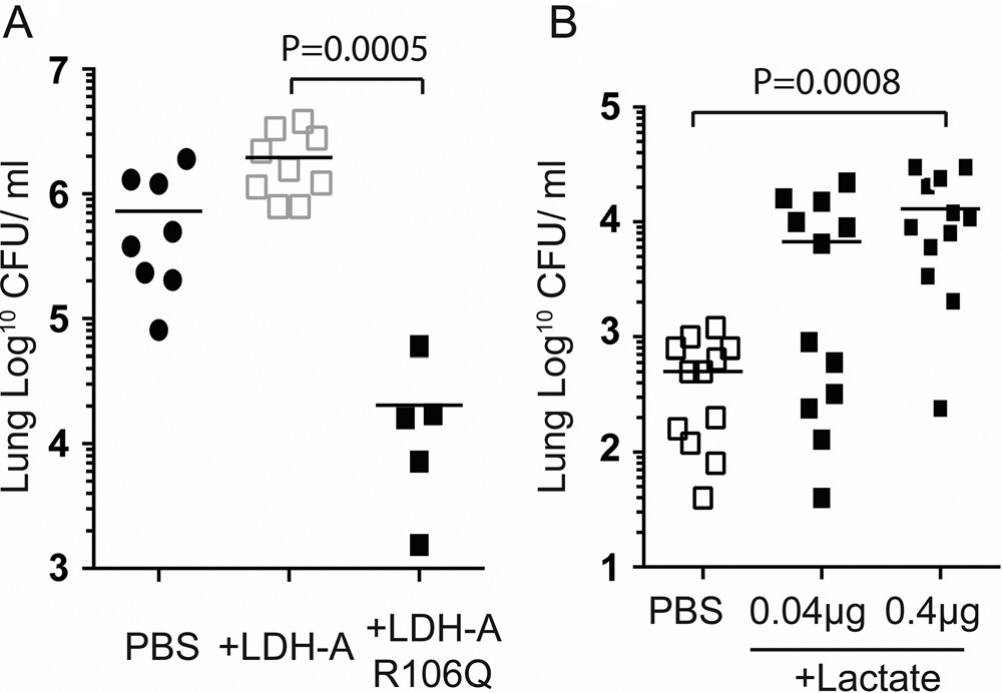
LDH-A activity promotes pneumococcal virulence. (A) Mice were infected intratracheally with 5.0 × 10^6^ CFU of WU2 alongside recombinant LDH-A or nonfunctional point mutated LDH-A (R106Q). (B) Mice were infected intratracheally with 1.0 × 10^6^ CFU EF3030 alongside different concentrations of lactate. Shapes in panels represent bacterial burden for each mouse. Statistical analyses were performed using a Kruskal-Wallis test with Dunn’s multiple-comparison posttest.

We tested and determined that LDH-A binding did not impact *Spn* adhesion or invasion of healthy lung epithelial cells grown *in vitro* (see Fig. S5). We also found no impact of PspA binding on LDH-A enzymatic activity *in vitro* (see Fig. S6). Yet, pneumococci incubated with enzymatically inactive LDH-A, as result of a point mutation (25), failed to enhance *Spn* virulence in mouse challenge experiments (Fig. 6A; see also Fig. S7). We interpreted the latter finding as suggesting LDH-A confers its impact on *Spn* virulence via the generation of lactate or NAD^+^. An important role for exogenous lactate was confirmed by our observation of increased *Spn* burden in the lungs of mice challenged with *Spn* suspended in saline with lactate versus *Spn* in saline alone (Fig. 6B). In contrast, no effect on lung bacterial burden was seen when *Spn* was mixed with saline containing exogenous NAD prior to challenge (NAD 100 μM, 2.03 ± 0.26 × 10^6^ CFU/g; control, 2.01 ± 0.46 × 10^6^ CFU/g; ± standard error of the mean [SEM]; *P* = 0.979, *n* = 7/cohort). Finally, recent findings by Minhas et al. demonstrated that endogenous generation of NAD^+^ is vital to macrophage immune function (26). We therefore tested whether treatment with exogenous NAD had an influence on the cytokine and chemokine response of J774A.1 macrophages challenged with *Spn* or on their ability to take up *Spn*. We observed no difference in the production of tumor necrosis factor (NAD, 400 ± 11.6; control, 455.9 ± 45.2; ± SEM; *P* = 0.4206, *n* = 5/cohort) by infected macrophages that had been treated with NAD or evidence that pretreatment with exogenous NAD enhanced efficiency of bacterial uptake by macrophages after 1 h of coincubation (NAD, 3.7% ± 1.7% of initial inoculum; control, 3.4% ± 1.2% [SEM]; *P* > 0.999, *n* = 5/cohort). From these data, we conclude the observed contribution of PspA to virulence was most likely the result of co-opting host LDH-A enzymatic activity and the generation of lactate.

10.1128/mBio.00673-21.7 FIG S5 LDH does not affect *Spn* adhesion and invasion. Preincubated *Spn* WU2 or EF3030-04 with LDH was incubated with A549 cells for 1 h and then washed (adhesion) 3 times by PBS or changed to DMEM medium with ampicillin-streptomycin (invasion) and incubated for 2 h and then washed 3× with PBS. Subsequently, A549 cells were detached by trypsin-EDTA, and then total bacterial CFU were calculated on blood agar plates (*n* = 3). Means and standard errors are shown. Download FIG S5, PDF file, 0.07 MB. Copyright © 2021 Park et al. 2021 Park et al. https://creativecommons.org/licenses/by/4.0/ This content is distributed under the terms of the Creative Commons Attribution 4.0 International license.

10.1128/mBio.00673-21.8 FIG S6 PspA does not affect LDH enzyme activity. Enzyme activity of preincubated LDH with different rations of PspA_WU2_ was measured using a LDH assay kit (*n* = 5). Means and standard errors shown. Download FIG S6, PDF file, 0.03 MB. Copyright © 2021 Park et al. 2021 Park et al. https://creativecommons.org/licenses/by/4.0/ This content is distributed under the terms of the Creative Commons Attribution 4.0 International license.

10.1128/mBio.00673-21.9 FIG S7 Nonfunctional point mutant LDH loses enzyme activity. Enzyme activity of LDH-A or nonfunctional point mutant LDH-A (R106Q) was measured using an LDH assay kit (*n* = 3). Means and standard errors shown. Download FIG S7, PDF file, 0.03 MB. Copyright © 2021 Park et al. 2021 Park et al. https://creativecommons.org/licenses/by/4.0/ This content is distributed under the terms of the Creative Commons Attribution 4.0 International license.

## DISCUSSION

PspA and PspC are multidomain CBPs with clear and vital roles in pneumococcal pathogenesis. Prior to this report, they had been demonstrated to confer resistance to opsonization by various means (27, 28). The vital role of these proteins in pneumococcal biology is highlighted by the fact that most clinical isolates of *Spn* carry both proteins (5, 29, 30), and mutants deficient in PspA and PspC are markedly reduced in virulence in a variety of experimental models (31–34). PspA and PspC are among the most common and highly *in vivo*-expressed CBPs (5, 14, 29, 35, 36), and immunization with these proteins as antigens has been demonstrated to confer protection against invasive pneumococcal disease, especially when used in combination (31, 37, 38). Nonetheless, and despite >30 years of study on PspA and PspC, prior to this report, the role of the NPB in pneumococcal biology was undetermined. This report assigns function to group 3 PRD, which houses the NPB, and shows that it enhances the bacterium’s virulence as result of binding to enzymatically active LDH-A from host cells. The role of group 1 and group 2 PRDs remain an open question, although immunity against the proline-rich portion of the PRD appears to be protective (14, 15).

LDH, NADH, and the substrate pyruvate have all been shown to be released by host cells when they die (22, 39). LDH-A converts pyruvate to lactate and, in doing so, generates NAD^+^ from NADH. NAD^+^ is an important redox carrier and serves as the sole substrate for NAD-dependent enzymes (40). Thus, its sustained generation by LDH-A has the potential to catalyze other enzymatic events at the infection site to the bacterium’s benefit that were not tested. For example, multiple NAD^+^-dependent antioxidants are produced by the host (41). Sustained NAD^+^ production has the potential to benefit *Spn* by enhancing the neutralization of the profuse amounts of H_2_O_2_ produced as result of *Spn*’s own metabolism (23). In addition, exogenous NAD has also been shown to alter T-cell function and, in certain instances, induce T-cell death (42, 43). Exposure to exogenous NAD has also been shown to enhance Fcγ receptor-mediated endocytosis by macrophages (44). Thus, co-opted LDH-A activity may not exclusively benefit *Spn*. Importantly, and as our mice were naive to *Spn* and therefore lacked antibodies or pathogen-specific T cells against *Spn*, our experimental model does not reflect the potential impact of NAD^+^ generated by LDH-A on these adaptive immune system parameters. This is an important area to explore in the future, particularly with consideration that antibodies generated against PspA may block its binding to LDH-A altogether during *Spn* reinfection.

Incubation of *Spn* with lactate was, on its own, sufficient to confer virulence but only if the *Spn* carried a PspA with an NPB. Consistent with this, there are numerous ways exogenous lactate production might benefit *Spn*. Pneumococcal lactate oxidase converts lactate to pyruvate, and the latter has been demonstrated to be a vital energy source for *Spn* during aerobic growth (45, 46). As both the nasopharynx and the lungs are aerobic sites, PspA binding to host LDH may be a way for the bacterium to directly augment its energy supply. Binding of the bacterium to host cells via PspC’s other binding domains would, in this scenario, also enhance *Spn*’s ability to harvest pyruvate from damaged cells before it is diluted in the milieu. Lactate also has immunomodulatory properties which may benefit the bacterium. For example, lactate has been shown to reduce organ injury during Toll-like receptor and inflammasome-mediated inflammation of the gut. This was as a result of its interaction with G_i_ protein-coupled receptor GPR81 on host cells (47). Notably, Toll-like receptor and inflammasome activation are the principal ways by which the host detects *Spn* in the airway, and GPR81 is also present on lung epithelial cells (48, 49). Critically, the above described are speculation, and detailed investigative studies on how lactate generation helps *Spn* are now warranted to fully understand PspA’s contribution to the disease process.

Despite this lack of clarity on how LDH-A confers a beneficial effect to *Spn*, that PspA can enhance *Spn* virulence in the airway by binding to LDH-A is unequivocal. Our strongest evidence for this being that EF3030, a strain that lacks the NPB in either its PspA or PspC, had increased virulence when mutated to carry a version of PspA with the NPB. Likewise, that deletion of only the NPB from WU2 PspA starkly reduced virulence. The fact that LDH is released from dying cells and enhances pneumococcal virulence may be important under several other conditions known to enhance susceptibility to *Spn*. These include but are not limited to secondary infections to influenza virus and the increase in *Spn* susceptibility associated with smoking, cancer, and chronic obstructive pulmonary disease, all of which have been associated with increased LDH in BALF (50, 51). Importantly, LDH production is host-cell-type-dependent and affected by levels of glucose in the cell’s environment (52, 53). Thus, its contribution to *Spn* pathogenesis may be anatomical site specific or even change over the course of pneumonia, as capillary leakage increases glucose levels in the airway (54). Finally, it should be noted that PRD and/or other parts of PspA or PspC may bind to other metabolism-associated host factors, and these interactions may also influence pneumococcal pathogenesis. This would not be unexpected as PspA and PspC are on the bacterial surface and host metabolic enzymes are known to be released in large quantity by host cells under a variety of conditions (22, 55).

In summary, we have identified a role for the NPB carried by the version of the PRD that is most often found in both PspA and PspC. That role is binding to LDH-A. PspA binding to LDH-A was shown to enhance the bacterium’s virulence during pneumonia. To confer this phenotype, LDH-A must be enzymatically active, with local generation of lactate having beneficial effects for the bacterium. Our findings suggest *Spn* utilizes host proteins released from dying cells to its advantage and highlight the complex interactions between *Spn* and its host during disease. Finally, the fact that some pneumococci have versions of PspA without an NPB may indicate they have compensating virulence functions or, perhaps more likely, that different pneumococci have different host interaction strategies that allow them to survive and pass to a subsequent new host.

## MATERIALS AND METHODS

### Bacteria, cloning, and plasmids.

All bacterial strains, plasmids, and primers used in this study are listed in Table S2 in the supplemental material. *Spn*, S. aureus, and uropathogenic Escherichia coli (UPEC) were cultured on blood agar plates at 37°C within a candle jar (*Spn*) or in a 5% CO_2_ incubator. All three *Spn* strains have PRD in their PspA. Colony swabs were used to inoculate Todd-Hewitt broth (THB) with 0.5% yeast extract and grown until mid-exponential phase (optical density at 621 nm [OD_621_] of 0.3). Isogenic *Spn* mutants deficient in *pspA* were generated by insertion of a kanamycin resistance cassette using standard homologous recombination methods (56). Briefly, DNA corresponding to the flanking regions of *pspA* were amplified by PCR (primers listed in Table S2) and cloned adjacent to *erm* and *kan* (56) in pCR2.1 (pCR2.1 *erm* and pCR2.1 *kan*). This mutagenic construct was PCR amplified and used to transform *Spn*. Hybrid versions of *pspA* were generated using a PCR-based fusion method using primers in Table S2. In this instance, the mutagenic DNA products were cloned into pCR2.1 *ermB* (57) and used to transform the aforementioned *pspA* kanamycin-resistant isogenic mutant. In both instances, mutagenic *Spn* isolates were selected on blood agar plates with antimicrobials and validated by sequencing of amplified DNA. Human recombinant LDH-A and LDH-B expression vectors were purchased from Applied Biological Material (Richmond, BC, Canada). We removed the encoded N-terminal glutathione transferase (GST) tag on both LDH-A and LDH-B using restriction enzymes and cloned the genes into the pTEV5 expression vector, which added a removable version of the His tag (kindly provided by Michael Gray, The University of Alabama at Birmingham).

10.1128/mBio.00673-21.2 TABLE S2 List of bacterial strains, plasmids, and primers used for this study. Download Table S2, PDF file, 0.05 MB. Copyright © 2021 Park et al. 2021 Park et al. https://creativecommons.org/licenses/by/4.0/ This content is distributed under the terms of the Creative Commons Attribution 4.0 International license.

### Expression and purification of recombinant proteins.

All protein expression vectors were transformed individually into E. coli NEBExpress *Iq* (New England BioLabs, Ipswich, MA) competent (PspA_WU2_ and PspA fragments) or E. coli BL21 for protein expression. After cultures reached on OD_600_ of 0.4 to 0.6, they were induced using 1 mM isopropyl-β-d-thiogalactopyranoside (IPTG) for 4 h at 37°C on shaker. Bacteria were harvested by centrifugation at 4,000 × *g* for 15 min. Cell pellets were resuspended in buffer A (50 mM Tris-HCl [pH 7.5] and 150 mM NaCl) with 1 mM phenylmethylsulfonyl fluoride (PMSF), a serine protease inhibitor, and then sonicated at 35% amplitude (2 s on/2 s off) for 30 min on ice for lysis and centrifuged at 12,000 × *g* for 30 min on 4°C. Overexpressed protein in the supernatant was purified using a cobalt resin column according to the manufacturer’s protocols for His tag purification. For LDH-A and LDH-B, the His tags were removed using tobacco etch virus protease and passage of the protein through a cobalt resin column to remove uncut protein. Purified proteins were loaded on the Superdex S200 or S75 columns using PBS. Collected protein fractions were checked by SDS-PAGE and concentrated by a centrifugal concentrator tube. The concentration of proteins was measured by UV 280 nm, and they were stored in Dulbecco’s phosphate-buffered saline (PBS) with 20% glycerol at −70°C.

### Cell lines and growth conditions.

Low-passage-number stocks of THP-1, a human monocyte cell line (58), and A549, a human type II pneumocyte cell line (59), were obtained from ATCC and cultured as recommended. Cell lysates from monolayers at ∼80% confluence were generated using RIPA buffer (150 mM NaCl, 50 mM Tris-HCl, 1% Triton X-100, 0.5% sodium deoxycholate, and 0.1% sodium dodecyl sulfate).

### Ni-NTA pulldown assay.

Two micrograms of recombinant human lactoferrin (OPSA11754; AVIVA Systems Biology, San Diego, CA) and rabbit muscle LDH (LDH-A) (Sigma, St. Louis, MO) were mixed with 2 μg of 6×His-PspA_WU2_, fragments (F-1, F2, F-3, F-4, or F-5), or recombinant 6×His-PRDs (WU2PRD, TIGR4 PRD, or EF6796 PRD) in buffer A (PBS, 0.1% Triton X-100, 0.5 mM dithiothreitol [DTT], and 10 mM imidazole) and then incubated for 1 h at 4°C on rotator (6 rpm). Nickel-nitrilotriacetic acid (Ni-NTA) resin (Qiagen), 20 μl, was equilibrated in 1 ml of buffer A. Subsequently, Ni-NTA resin and mixtures of proteins were mixed together and incubated for 1 h at 4°C on a rotator (6 rpm). Incubated Ni-NTA resins were washed four times with 1 ml of buffer A containing 20 mM imidazole. SDS loading buffer (2×) was added to the Ni-NTA resins and then incubated at 90°C for 5 min. Proteins were separated on 4 to 12% gradient SDS-PAGE gels (Invitrogen) and visualized by Coomassie blue staining or immunoblots with an anti-LDH antibody (ab130923; Abcam).

### ELISA.

Microtiter 96-well plates (Nunc, Weisbaden, Germany) were coated overnight at 4°C with 100 μl of different concentrations (0 to 4 μg/well) of recombinant human LDH-A, recombinant human LDH-B, or recombinant *Spn* PspA in PBS. Plates were washed, blocked with 200 μl 5% bovine serum albumin (BSA) in PBS for 2 h, washed three times by T-PBS (PBS with 0.5% Tween 20), and then incubated with 2 μg/well of PspA_WU2_ or A549 lysate at 37°C for 1 h. The plate was washed three times with T-PBS. Finally, the plate was incubated at room temperature for 1 h with mouse monoclonal anti-PspA (1b2.21) (60) or rabbit monoclonal anti-LDH (ab130923) antibody (1:1,000) in PBS with 5% BSA, washed three times with T-PBS, incubated for 1 h with 1:10,000 alkaline phosphatase-conjugated donkey anti-rabbit immunoglobulin antiserum IgG(H+L) (Southern Biotechnology Associates, Birmingham, AL), washed, and developed with *p*-nitrophenyl phosphate (Sigma) or 3,3′,5,5′ tetramethylbenzidine (TMB; BD Biosciences); the absorbance was read at 405 nm or 450 nm.

### Detection of bound FITC-LDH on bacteria.

Pneumococci, S. aureus, or urinary tract-pathogenic E. coli isolates were grown as described above and resuspended to 1.0 × 10^8^ CFU/ml in fluorescence-activated cell sorting (FACS) buffer (1× PBS with 3% fetal bovine serum [FBS] and 0.1% sodium azide). For the LDH binding assay, 1.0 × 10^6^ CFU bacteria were incubated in 100 μl with 0.5 μg of FITC-conjugated LDH at 4°C for 30 min with shaking. Samples were washed 2 times with FACS buffer and then analyzed by flow cytometry using BD Accuri C6 (BD, Franklin Lakes, NJ).

### LDH measurement.

Cell death was evaluated by detection of lactate dehydrogenase (LDH) in culture supernatants as previously described (61). For measurement of LDH *in vivo*, 6- to 8-week-old C57BL/6 mice were infected with *Spn* TIGR4 (5 × 10^5^ CFU) in 40 μl saline by forced aspiration. One day after challenge, mice were euthanized, and then bronchoalveolar lavage (BAL) was performed using 1 ml of ice-cold PBS. Fifty microliters of BAL fluid (BALF) per mouse was transferred to a flat-bottom 96-well plate and used for detection of LDH. LDH in supernatants and BALF was measured using a Pierce LDH cytotoxicity assay kit (Thermo Fisher Scientific, Suwanee, GA). Plates were read using an iMark absorbance microplate reader (Bio-Rad Laboratories, Hercules, CA).

### Immunoblotting.

Cultured *Spn* isolates were resuspended by RIPA buffer (Sigma) with proteinase inhibitor cocktail (Sigma) and lysed by the freeze-thaw method. Supernatants were collected, and the protein concentration was measured by bicinchoninic acid assay (Bio-Rad, Hercules, CA). Cell lysates (10 μg) were run on SDS-PAGE gels, and gels were electroblotted to a 0.45-μm-pore-size nitrocellulose membrane (Bio-Rad). The membrane was blocked with 3% bovine serum albumin in T-PBS (PBS containing 0.05% Tween 20) for 1 h at room temperature. The membranes were exposed to anti-NPB antibody (PR-1A4.7) (15) or anti-PspA antibody (1b2.21) for 1 h at room temperature, washed, and incubated with horseradish peroxidase (HRP)-conjugated goat anti-mouse IgG (diluted 1:10,000 in PBS-T) for 1 h at room temperature. After washing, the membranes were developed with enhanced chemiluminescence (ECL) solution (Bio-Rad) and observed by using a ChemiDoc (Bio-Rad).

### Mouse challenge.

Female 8- to 12-week-old BALB/c mice (The Jackson Laboratory) were used for protection studies. Briefly, *Spn* strains WU2, TIGR4, and EF3030 were incubated in THB until an OD_621_ of 0.3 (∼1.0 × 10^8^ CFU/ml) and resuspended in PBS with or without of 5 μg/mouse LDH. Unbound LDH on EF3030 was removed by centrifugation, and the bacteria were resuspended in PBS. The mice were infected by forced aspiration with 40 μl of *Spn* WU2 (5.0 × 10^6^ CFU), TIGR4 (1.0 × 10^5^ CFU), or EF3030 (1.0 × 10^6^ CFU). After 24 h, mice were euthanized, and then total CFU in the lungs were measured by serial dilution of homogenized lungs and extrapolation of colony counts on blood agar plates the next day.

### Macrophage response assays.

The J774.1 macrophage cell line was seeded in a 12-well plate with 5 × 10^5^ cells and grown in Dulbecco’s modified Eagle medium (DMEM) for 2 days. Cells were treated 1 h prior to infection with either medium alone or medium plus NAD at 50 μM. Cells were then washed with PBS three times to remove medium. Cells were then inoculated with Spn strain TIGR4 at a multiplicity of infection (MOI) of 10 (1 × 10^7^) and spun down at 300 × *g* for 5 min to synchronize infection. After 1 h, the supernatant was collected for tumor necrosis factor (TNF) (DY-410) ELISA from R&D. Macrophages were washed and lysed with water, and associated *Spn* cells were enumerated for extrapolation of phagocytosis efficiency.

### Statistical analyses.

Statistical analyses were performed using GraphPad Prism 6 software using nonparametric Mann-Whitney U test, Kruskal-Wallis test with Dunn’s multiple-comparison posttest, or two-way ANOVA.

10.1128/mBio.00673-21.10 TEXT S1 Supplemental methods. Download Text S1, PDF file, 0.10 MB. Copyright © 2021 Park et al. 2021 Park et al. https://creativecommons.org/licenses/by/4.0/ This content is distributed under the terms of the Creative Commons Attribution 4.0 International license.
