# Supplementary material for: Streptococcus pneumoniae Binds to Host Lactate Dehydrogenase via PspA and PspC To Enhance Virulence
Source: mBio. 2021 May 4;12(3):e00673-21. doi: 10.1128/mBio.00673-21 (PMC8437407; doi:10.1128/mBio.00673-21)
Supplement: TABLE S1 [file mbio.00673-21-st001.pdf]

Table S1. List of PspA interacted THP-1 proteins

Accession	Description	# PSMs
P08670	Vimentin OS=Homo sapiens OX=9606 GN=VIM PE=1 SV=4	1381
P02545	Prelamin-A/C OS=Homo sapiens OX=9606 GN=LMNA PE=1 SV=1	905
**P04406**	**Glyceraldehyde-3-phosphate dehydrogenase OS=Homo sapiens OX=9606 GN=GAPDH PE=1 SV=3**	**583**
P23246	Splicing factor, proline- and glutamine-rich OS=Homo sapiens OX=9606 GN=SFPQ PE=1 SV=2	282
**P00338**	**L-lactate dehydrogenase A chain OS=Homo sapiens OX=9606 GN=LDHA PE=1 SV=2**	**277**
