# Supplementary material for: Streptococcus pneumoniae Binds to Host Lactate Dehydrogenase via PspA and PspC To Enhance Virulence
Source: mBio. 2021 May 4;12(3):e00673-21. doi: 10.1128/mBio.00673-21 (PMC8437407; doi:10.1128/mBio.00673-21)
Supplement: FIG S1 [file mbio.00673-21-sf001.pdf]

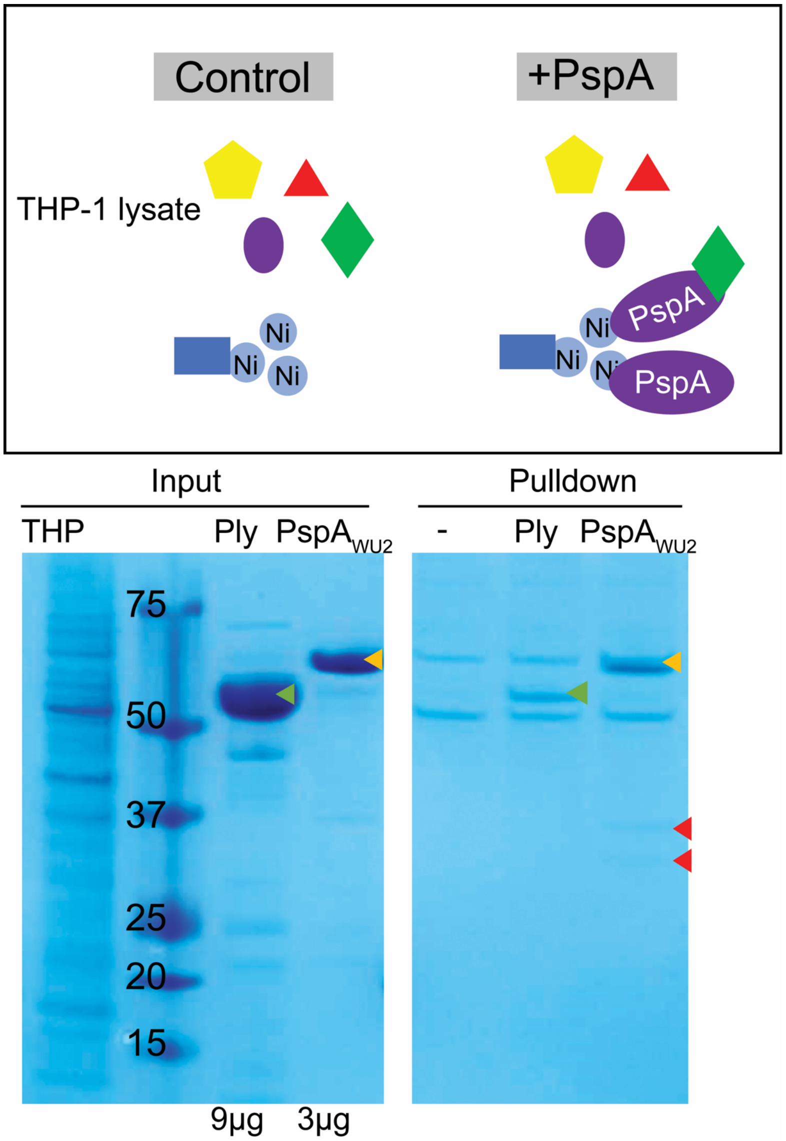

Figure S1. Purified PspA binds to host LDH and GAPDH. Recombinant His-tagged pneumolysin (Ply, green arrow) or PspA$_{WU2}$ (yellow arrow) were pulled-down with human macrophage THP-1 cell lysate using Ni-NTA resin. The input and bound proteins were separated by SDS-PAGE and visualized by Coomassie blue dye. PspA bound host proteins (red arrows) were listed on Table S1.
