# Supplementary material for: Streptococcus pneumoniae Binds to Host Lactate Dehydrogenase via PspA and PspC To Enhance Virulence
Source: mBio. 2021 May 4;12(3):e00673-21. doi: 10.1128/mBio.00673-21 (PMC8437407; doi:10.1128/mBio.00673-21)
Supplement: FIG S2 [file mbio.00673-21-st002.pdf]

Table S2. Bacteria species and strains for this study

Strain	Capsular type	Mutation	Reference
Streptococcus pneumoniae			
WU2	3	Clinical isolated	(6)
WU2*ΔpspA kam*^{R}		Isogenic deletion mutant of full length gene of *pspA*, *kam*^{R}	This study
WU2*ΔpspA*		Isogenic deletion mutant of full length gene of *pspA*, *erm*^{R}	This study
WU2*ΔNPB*		Isogenic deletion mutant of NPB on pspA, *erm*^{R}	This study
TIGR4	4	Clinical isolated	(7)
EF3030	19F	Clinical isolated	(8)
EF3030 *ΔpspA kam*^{R}		Isogenic deletion mutant of full length gene of *pspA*, *kam*^{R}	This study
EF3030-01		Isogenic deletion mutant using *erm*^{R}	This study
EF3030-04		Isogenic deletion mutant, replacement pspA from WU2, *erm*^{R}	This study
EF3030-05		Isogenic deletion mutant, replacement pspA without *NPB* from WU2, *erm*^{R}	This study
EF3296	4	Clinical isolated	(8)
DBL1	6B	Clinical isolated	(9)
L81905	4	Clinical isolated	(9)
E134	23	Clinical isolated	(10)
BG11703	18	Clinical isolated	(8)
RX1	Non-encapsulated		(11)
BG7817	12	Clinical isolated	(8)
AC94	9	Clinical isolated	(8)
BG6380	37	Clinical isolated	(8)
BG8743	23	Clinical isolated	(8)
Escherichia coli			
NEB®Express *I*^{q} Competent *E. coli*		*MiniF lacI*^{q}*(Cam*^{R}*) / fhuA2 [lon] ompT gal sulA11 R(mcr-73::miniTn10--Tet*^{S}*)2 [dcm] R(zgb-210::Tn10--Tet*^{S}*) endA1 Δ(mcrC-mrr)114::IS10*	NEB

BL21(DE3)		*gal (cIts857 ind1 Sam7 nin5 lacUV5-T7 gene 1)*	Novagen
XL1-Blue		*recA1 endA1 gyrA96 thi-1 hsdR17 supE44 relA1 lac [F' proAB lacI ^{q} Z DM15 Tn10 (Tet^r)]*	Novagen
**Other Pathogens**			
Staphylococcus aureus		Clinical isolate	(12)
Uropathogenic *E. coli*		Clinical isolate	(12)

Table S2. List of plasmids and primers for this study

Name	Relevant characteristics	Refence or source
**Plasmids**		
pCR2.1 *erm*^{R}	pCR2.1, contained *erm*^{R} cassette	(13)
pCR2.1 *kan*^{R}	pCR2.1, contained *kan*^{R} cassette	This study
pQE-SS01 (F-1)	T5 promotor, N−terminal his-tagged fragment of $PspA_{WU2}$(32-317aa)	This study
pQU-SS02 ($PspA_{WU2}$)	T5 promotor, N−terminal his-tagged $PspA_{WU2}$(32-417aa)	This study
pQE-SS10 (F-2)	T5 promotor, N−terminal his-tagged fragment of $PspA_{WU2}$(32-281aa)	This study
pQE-SS11 (F-3)	T5 promotor, N−terminal his-tagged fragment of $PspA_{WU2}$(32-229aa)	This study
pQE-SS12 (F-4)	T5 promotor, N−terminal his-tagged fragment of $PspA_{WU2}$(32-200aa)	This study
pQE-SS13 (F-5)	T5 promotor, N−terminal his-tagged fragment of $PspA_{WU2}$(32-148aa)	This study
pQE-SS04 (WU2PRD)	T5 promotor, N−terminal his-tagged fragment of $PspA_{WU2}$(318-417aa)	This study
pET32b-TIGR4 PRD	N−terminal thioredoxin and his-tagged TIGR4 PRD	(14)
pQE-EF6796 PRD	T5 promotor, N-terminal his tagged EF6796 PRD	This study
LDHB protein vector (human)	pPB-His-GST (PV375851)<br>contained N-terminal Nhe1 site, C-terminal Xho1 site	Applied biological material
LDHA protein vector (human)	pPB-His-GST (PV375815)<br>contained N-terminal Nhe1 site, C-terminal Xho1 site	Applied biological material
pTEV5	N-terminal his-tag and TEV recognition site	(15)
pTEV5-LDHA	pTEV5-human LDHA from LDHA protein vector	This study
pTEV5-LDHB	pTEV5-human LDHB from LDHB protein vector	This study
**Primers**		

LDHA R106Q Fw	5’-AAGAGGGAGAAAGCCAGCTTAATTTGGTCCAG
LDHA R106Q Rw	5’-CTGGACCAAATTAAGCTGGCTTTCTCCCTCTT
delNPB-MF	5’-CAACCCAAACCAGCTCCAGCAGAAAAACCAGCTCCAGCACC
delNPB-MR	5’-GGTGCTGGAGCTGGTTTTTCTGCTGGAGCTGGTTTGGGTTG
pspA-F	5’-CGCGGATCCAAGCTTATGATATAGAAATTTGTAAC
pspA-R	5’-GAGGAGCTCTTAAACCCATTCACCATTGGC
EF6796 Fw	5’-CCTTAAGAAAGCAGTTGGATCCCCAGAAAC
EF6796 Rw	5’-CCGTTTTCTTGTTAAGCTTATATTTTTGG
PspA$_{WU2}$ (31-)-F	GTAGGATCCGAAGAATCTCCCGTAGC
PspA$_{WU2}$ (-417)-R	CCGTGGTACCGTTTCCATCATGTTTT
PspA$_{WU2}$ (-317)-R	CTGAGGTACCTCACTCATTAACTGCTTTCTT
PspA$_{WU2}$ (-317)-F	CCTTAAGAAAGCAGTTGGATCCCCAGAAA
PspA$_{WU2}$ (-281)-R	ACCCGGGTACCTCATTCTTCAGCAGCTTTAAG
PspA$_{WU2}$ (-229)-R	ACCCGGGTACCTCATTCTTCAGCATCCACTTTTTG
PspA$_{WU2}$ (-200)-R	ACCCGGGTACCTCATGATTCAGACTCATCAATCTC
PspA$_{WU2}$ (-148)-R	ACCCGGGTACCTCAAACTACCATTGTTGTTCGAATAGT
PspA-L-F	GCTCTAGAAATATAAAGTATAGTCTAAAAATGA
PspA-L-R	ATAAGAATGCGGCCGCAACAAAACCAGCCCC
PspA-R1-F	CGCGGATCCGGTGAATGGGTTTAAGCCG
PspA-R1-R	CCCAAGCTTCAACCCATTTTCTCTAATC
kan^{R}-cassette-F	ATAAGAATGCGGCCGCGCAAGGAACAGTGAATTGGA
kan^{R}-cassette-R	CGGAATTCCAGTTGCGGATGTACTTCAG
PspA-R2-F	GGACTAGTAGCCGATTAAATT

PspA-R2-R	CGGGATCCCGTCCGGATTTGGCG
