# Supplementary material for: Streptococcus pneumoniae Binds to Host Lactate Dehydrogenase via PspA and PspC To Enhance Virulence
Source: mBio. 2021 May 4;12(3):e00673-21. doi: 10.1128/mBio.00673-21 (PMC8437407; doi:10.1128/mBio.00673-21)
Supplement: FIG S3 [file mbio.00673-21-sf003.pdf]

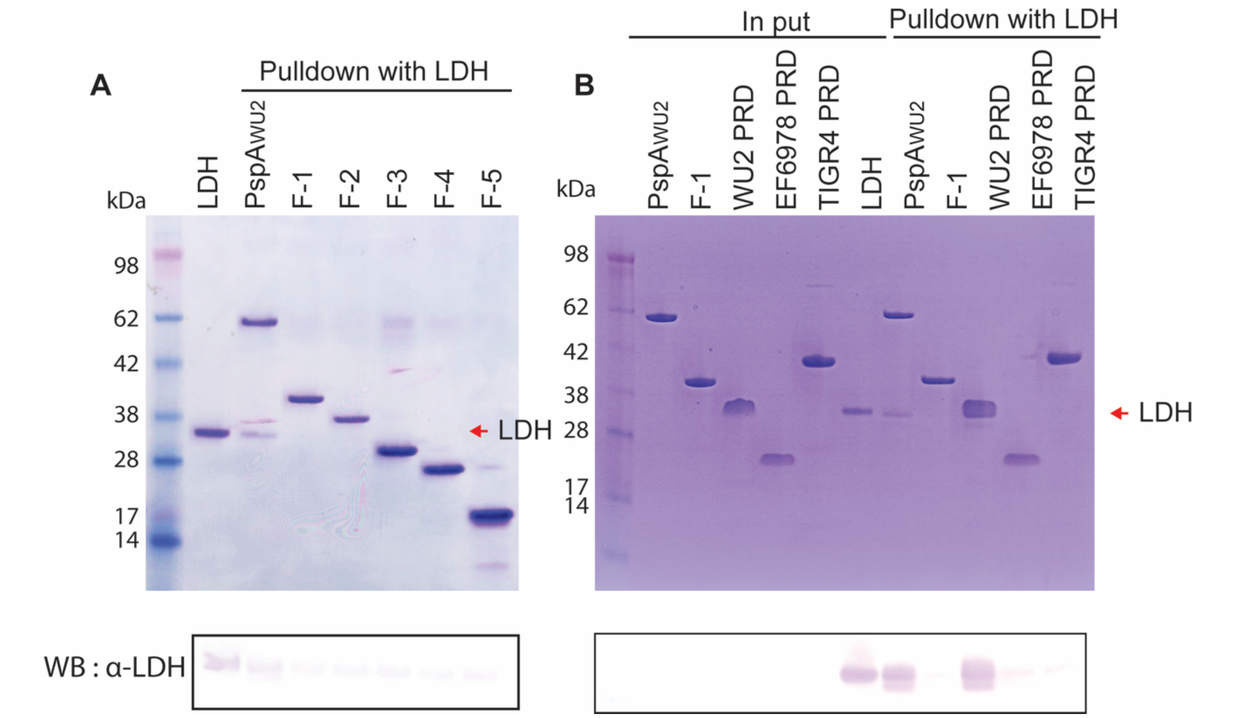

Figure S3. LDH binds to Group 3 PRD motif. (A) $PspA_{WU2}$, fragments (F-1, F-2, F-3, F-4 and F-5), (B) PRDs (WU2 PRD, EF6978 PRD and TIGR4 PRD) as bait were pulled-down with LDH and as visualized by Coomassie blue stains. Bound LDH were detected by immunoblotting using monoclonal anti-LDH antibody. The red arrow indicated LDH size.
