# Supplementary material for: Streptococcus pneumoniae Binds to Host Lactate Dehydrogenase via PspA and PspC To Enhance Virulence
Source: mBio. 2021 May 4;12(3):e00673-21. doi: 10.1128/mBio.00673-21 (PMC8437407; doi:10.1128/mBio.00673-21)
Supplement: FIG S4 [file mbio.00673-21-sf004.pdf]

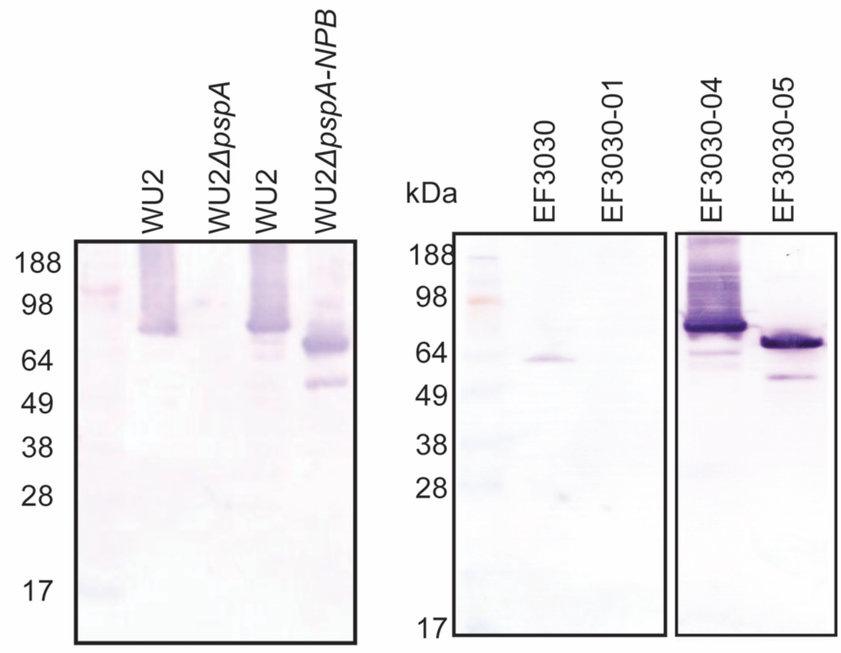

Figure S4. PspA patterns of WU2 and EF3030 wild type or different isogenic mutants. Total cell lysates of *Spn* WU2, EF3030, isogenic *pspA* mutants (WU2Δ*pspA* and EF3030-01), isogenic pspA mutants (WU2Δ*NPB*), EF3030 expressing $PspA_{WU2}$ (EF3030-04) and EF3030 expressing $PspA_{WU2}$ without NPB (EF3030-05) were electrophorized on SDS-PAGE. Subsequently, expressed PspA were detected by using monoclonal anti-PspA antibody.
