# Supplementary material for: Streptococcus pneumoniae Binds to Host Lactate Dehydrogenase via PspA and PspC To Enhance Virulence
Source: mBio. 2021 May 4;12(3):e00673-21. doi: 10.1128/mBio.00673-21 (PMC8437407; doi:10.1128/mBio.00673-21)
Supplement: FIG S5 [file mbio.00673-21-sf005.pdf]

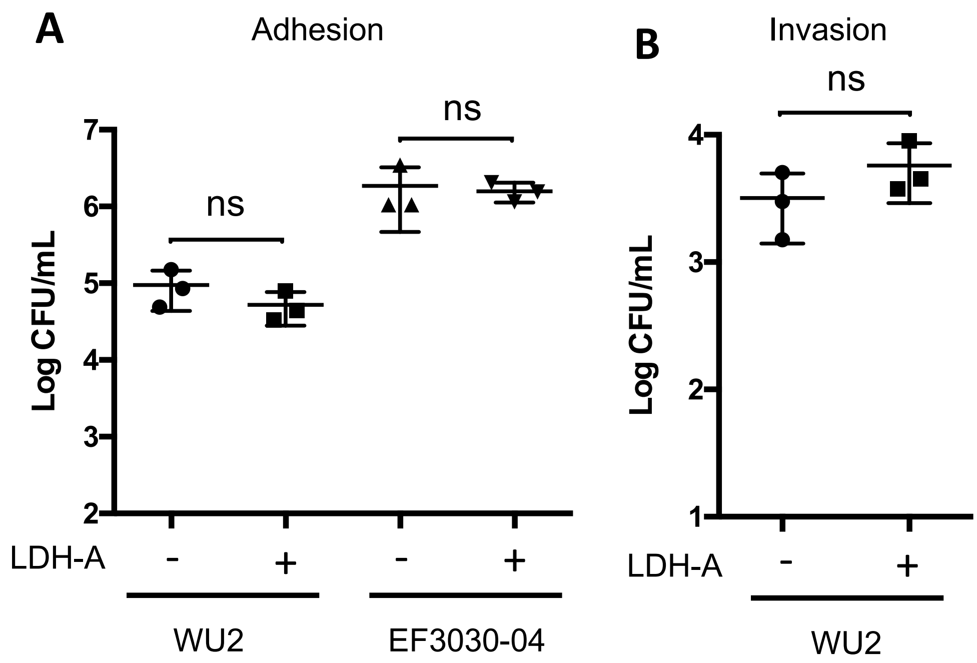

Figure S5. LDH does not affect *Spn* adhesion and invasion. Pre-incubated *Spn* WU2 or EF3030-04 with LDH were incubated A549 cell for 1 h and then washed (adhesion) 3 times by PBS or changed DMEM media with ampicillin/streptomycin (invasion) and incubated for 2 h and then washed by PBS 3X. Subsequently A549 cell were detached by Trypsin-EDTA and then total bacterial CFU were calculated on blood agar plate (N=3). Mean and standard error shown.
