# Supplementary material for: Streptococcus pneumoniae Binds to Host Lactate Dehydrogenase via PspA and PspC To Enhance Virulence
Source: mBio. 2021 May 4;12(3):e00673-21. doi: 10.1128/mBio.00673-21 (PMC8437407; doi:10.1128/mBio.00673-21)
Supplement: FIG S6 [file mbio.00673-21-sf006.pdf]

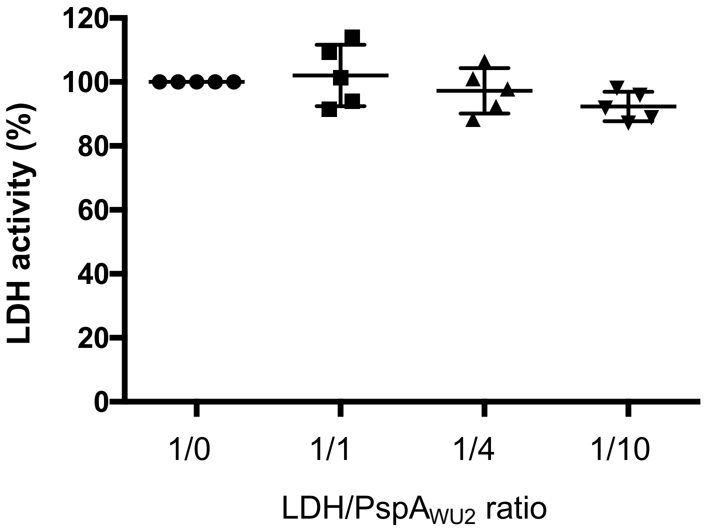

Figure S6. PspA does not affect LDH enzyme activity. Enzyme activity of pre-incubated LDH with different rations of $PspA_{WU2}$ were measured using a LDH assay kit (N=5). Mean and standard error shown.
