# Supplementary material for: Streptococcus pneumoniae Binds to Host Lactate Dehydrogenase via PspA and PspC To Enhance Virulence
Source: mBio. 2021 May 4;12(3):e00673-21. doi: 10.1128/mBio.00673-21 (PMC8437407; doi:10.1128/mBio.00673-21)
Supplement: FIG S7 [file mbio.00673-21-sf007.pdf]

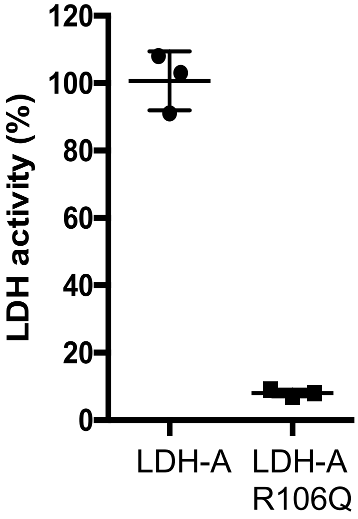

Figure S7. Non-functional point mutant LDH loses enzyme activity. Enzyme activity of LDHA or non-functional point mutant LDHA (R106Q) were measured using a LDH assay kit (N=3). Mean and standard error shown.
