# Supplementary material for: Streptococcus pneumoniae Binds to Host Lactate Dehydrogenase via PspA and PspC To Enhance Virulence
Source: mBio. 2021 May 4;12(3):e00673-21. doi: 10.1128/mBio.00673-21 (PMC8437407; doi:10.1128/mBio.00673-21)
Supplement: TEXT S1 [file mbio.00673-21-s0001.pdf]

SUPPLEMENTARY METHODS

Lactate dehydrogenase enzyme activity assay. Lactate dehydrogenase (LDH) enzyme activity was measured using an LDH Assay Kit (ab102526, Abcam). Briefly, t 0.5 μg of recombinant LDH-A and LDH-A with a R106Q deactivating point mutation was mixed with 50 μl of LDH assay buffer with or without $PspA_{WU2}$ and then reacted with 48 μl of assay buffer with 2 μl of substrate mix for 30min at room temperature. Changes in optical density (OD_{450} nm), which are indicative of LDH enzyme activity, were recorded every 2-3 minutes, for at least 30 minutes, using an iMark^{TM} Microplate absorbance reader (Bio-rad). LDH activity was calculated following kit protocol.

Cell lysate preparation and LC-MS/MS analysis.

THP-1 cell pellets were first rinsed with cold PBS to remove culture medium contamination. The pellet fraction was then lysed with 2 X SED lysis buffer followed by sonication in six 45 s on/off cycles while cooling the lysates in an ice-water bath. After a 10 min centrifugation step at 16,000 × g, the soluble lysate fraction was collected. Lysate corresponding to 0.8 mg of protein was incubated with 3 μg His-tagged $PspA_{WU2}$ or 9 μg His-tagged Ply for 2h at 4 °C. As a control, THP-1 lysates were loaded to the Ni-NTA resin alone to distinguish nonspecific interactions. Then, pull-down assay was done using an Ni-NTA affinity column (1). Bound proteins were eluted by boiling in 2 X NuPAGE LDS sample buffer for 5 min at 95°C. The eluted proteins were identified using mass spectrometry (2, 3). The LC–MS/MS analysis was conducted on an Ultimate 3000 RSLCnano System coupled to a quadrupole Orbitrap mass spectrometer (Q Exactive,

Thermo Scientific) via a nano electrospray ion source. The experimental methods were described previously in detail (4).

Protein identification and quantitation.

The MS raw data were searched against a meta database that contained protein sequences of *Homo sapiens* (UniProt taxon identifier 9606; 17,023 sequences). The MaxQuant-Andromeda software suite (version 1.6.5.0) was employed with most of the default settings (5). Two mis cleavage sites were allowed; the minimum peptide length was set to seven amino acids. Protein N-terminal acetylation and oxidation (M) and were set as variable modifications; carbamidomethylation (C) was set as fixed modification. The MS1 and MS2 ion tolerances were set at 20 ppm and 10 ppm, respectively. The false-discovery rate (FDR) of 1% was set at both peptide and protein level. The embedded label-free algorithm (MaxLFQ) was enabled using a minimum ratio count of 1. First, the proteins that were potential contaminants, identified in the decoy database or only by site modification were excluded for further analysis. The data were then log2 transformed and filtered to include those that were present in at least two of the three biological replicates in one of the two groups. The missing values were imputed based on default parameters. For hierarchical clustering, the Z-scored LFQ intensities were used with Euclidean as a distance measure for both column and row clustering.

Cell adhesion and invasion assay. *Spn* colonies on overnight plates were inoculated into the THY broth and cultivated at 37 °C until OD_{621} 0.3. The bacteria (~5.0 X 10^7 CFU) with or without addition of 20 μg of LDH was added to ~1.0 X 10^6 A549 cells (multiplicity

of infection ~50:1) in a 6-well polystyrene plate. *Spn* and cells were incubated for 30 min at 37 °C. Cells were washed with PBS 3X, and host cells along with attached bacteria were dislodged from the the wells by treatment with 0.25% trypsin and 0.02% EDTA for 5 min. The suspension was serially diluted and plated on 5% sheep blood agar plates. The number of attached bacteria were extrapolated from colony counts following incubation of the plates at 37 °C in a candle jar. For cell invasion, initial steps were the same as those used in the adhesion assay with exception that penicillin (100 U/ml) and Streptomycin (100 U/ml) were added to the culture media for 30 min following bacterial co-incubation with cells for 2 h at 37 °C to kill all non-internalized bacteria. Following 3X wash to remove residual antimicrobials, cells were harvested from the wells using 0.25% trypsin and 0.02% EDTA for 5 min. Extrapolation of invasion from colony counts was similar to the adhesion assay.

Fluorescein isothiocyanate conjugation.

Rabbit muscle LDH was conjugated by FluoroTag™ FITC-Conjugation Kit (Sigma) with slight modification to the manufacturer's recommendation. Briefly, 200 μl of LDH (1 μg/ ml) was mixed 50 μl of FITC (0.5 μg/ ml), 25 μl of PBS and 25 μl of 1 M sodium carbonate-bicarbonate pH 9.0 and incubated for 120 min or 30 min on shaker under the dark. Subsequently, conjugated proteins were dialyzed on PBS and then stored at -70 °C with 20% glycerol.

References

1. Liu C, Yu Y, Liu F, Wei X, Wrobel JA, Gunawardena HP, Zhou L, Jin J, Chen X. 2014. A chromatin activity-based chemoproteomic approach reveals a transcriptional repressome for gene-specific silencing. Nat Commun 5:5733.
2. Lin Y-H, Eguez RV, Torralba MG, Singh H, Golusinski P, Golusinski W, Masternak M, Nelson KE, Freire M, Yu Y. 2019. Self-Assembled STrap for Global Proteomics and Salivary Biomarker Discovery. Journal of Proteome Research 18:1907-1915.
3. Yu Y, Pieper R. 2019. Using Proteomics to Identify Inflammation During Urinary Tract Infection. Methods Mol Biol 2021:259-272.
4. HaileMariam M, Eguez RV, Singh H, Bekele S, Ameni G, Pieper R, Yu Y. 2018. S-Trap, an Ultrafast Sample-Preparation Approach for Shotgun Proteomics. Journal of Proteome Research 17:2917-2924.
5. Tyanova S, Temu T, Cox J. 2016. The MaxQuant computational platform for mass spectrometry-based shotgun proteomics. Nat Protocols 11:2301-2319.
6. Briles DE, Nahm M, Schroer K, Davie J, Baker P, Kearney J, Barletta R. 1981. Antiphosphocholine antibodies found in normal mouse serum are protective against intravenous infection with type 3 streptococcus pneumoniae. J Exp Med 153:694-705.
7. Tettelin H, Nelson KE, Paulsen IT, Eisen JA, Read TD, Peterson S, Heidelberg J, DeBoy RT, Haft DH, Dodson RJ, Durkin AS, Gwinn M, Kolonay JF, Nelson WC, Peterson JD, Umayam LA, White O, Salzberg SL, Lewis MR, Radune D, Holtzapple E, Khouri H, Wolf AM, Utterback TR, Hansen CL, McDonald LA, Feldblyum TV, Angiuoli S, Dickinson T, Hickey EK, Holt IE, Loftus BJ, Yang F, Smith HO, Venter JC, Dougherty BA, Morrison DA, Hollingshead SK, Fraser CM. 2001. Complete genome sequence of a virulent isolate of Streptococcus pneumoniae. Science 293:498-506.
8. Hollingshead SK, Becker R, Briles DE. 2000. Diversity of PspA: mosaic genes and evidence for past recombination in Streptococcus pneumoniae. Infect Immun 68:5889-900.
9. Briles DE, Crain MJ, Gray BM, Forman C, Yother J. 1992. Strong association between capsular type and virulence for mice among human isolates of Streptococcus pneumoniae. Infect Immun 60:111-6.
10. Brooks-Walter A, Briles DE, Hollingshead SK. 1999. The pspC gene of Streptococcus pneumoniae encodes a polymorphic protein, PspC, which elicits cross-reactive antibodies to PspA and provides immunity to pneumococcal bacteremia. Infect Immun 67:6533-42.
11. Shoemaker NB, Guild WR. 1974. Destruction of low efficiency markers is a slow process occurring at a heteroduplex stage of transformation. Mol Gen Genet 128:283-90.

12. Gonzalez-Juarbe N, Gilley RP, Hinojosa CA, Bradley KM, Kamei A, Gao G, Dube PH, Bergman MA, Orihuela CJ. 2015. Pore-Forming Toxins Induce Macrophage Necroptosis during Acute Bacterial Pneumonia. PLoS Pathog 11:e1005337.
13. Embry A, Hinojosa E, Orihuela CJ. 2007. Regions of Diversity 8, 9 and 13 contribute to Streptococcus pneumoniae virulence. BMC Microbiol 7:80.
14. Mukerji R, Hendrickson C, Genschmer KR, Park SS, Bouchet V, Goldstein R, Lefkowitz EJ, Briles DE. 2018. The diversity of the proline-rich domain of pneumococcal surface protein A (PspA): Potential relevance to a broad-spectrum vaccine. Vaccine 36:6834-6843.
15. Rocco CJ, Dennison KL, Klenchin VA, Rayment I, Escalante-Semerena JC. 2008. Construction and use of new cloning vectors for the rapid isolation of recombinant proteins from Escherichia coli. Plasmid 59:231-7.
